# A review of recent advances in the diagnosis of cardiac amyloidosis, treatment of its cardiac complications, and disease-modifying therapies

**DOI:** 10.12688/f1000research.130285.1

**Published:** 2023-02-20

**Authors:** Maharshi Raval, Sajid Siddiq, Kamal Sharma, Labdhi Sanghvi, Akhil Jain, Sagar Patel, Jaahnavee Trivedi, Kanishka Uttam Chandani, Dhriti Patel, Rupak Desai

**Affiliations:** 1 Department of Internal Medicine, New York Medical College, Valhalla, New York, USA; 2 Department of Internal Medicine, Landmark Medical Center, Woonsocket, Rhode Island, USA; 3 Department of Cardiology, New York Medical College, Valhalla, New York, USA; 4 Department of Cardiology, Landmark Medical Center, Woonsocket, Rhode Island, USA; 5 Department of Cardiology, B.J. Medical College and Civil Hospital, Ahmedabad, Gujarat, India; 6 Department of Pediatrics, Ahmedabad Municipal Corporation Medical Education Trust Medical College, Ahmedabad, Gujarat, India; 7 Department of Internal Medicine, Mercy Fitzgerald Hospital, Darby, Pennsylvania, USA; 8 Department of Medicine, B.J. Medical College and Civil Hospital, Ahmedabad, Gujarat, USA; 9 Independent Researcher, Atlanta, USA

**Keywords:** cardiac amyloidosis, heart failure, bone scintigraphy, ATTR amyloidosis, AL amyloidosis, tafamidis

## Abstract

Cardiac amyloidosis (CA), a significant condition resulting in infiltrative cardiomyopathy and heart failure with preserved ejection fraction (HFpEF), is caused by extracellular deposition of amyloid fibrils in the heart. Even though this has been known for an extended period, its prevalence in elderly patients with heart failure is increasingly being recognized. Recent advances in diagnosis with non-invasive methods like technetium pyrophosphate-labeled cardiac scintigraphy (i.e., Tc-PYP scan) and treatment options with tafamidis have played a pivotal role in awareness of the burden of this disease. Management of cardiac complications like heart failure, atrial arrhythmias, conduction block, ventricular arrhythmias, coronary artery disease, and aortic stenosis is now more critical than ever. We aim to review and outline the recent advances in diagnoses of CA. We also review management strategies for cardiac complications of CA with a brief summary of disease-modifying therapies.

## Introduction

Cardiac amyloidosis (CA) is primarily classified as either transthyretin (ATTR) or light chain (AL) amyloidosis. ATTR amyloidosis may result from a genetic mutation in the TTR gene, which is inherited (ATTRv), or may result from age-related deposition from wild-type ATTR (ATTRwt). ^1^^,^^2^

CA should be suspected in elderly patients with heart failure symptoms such as dyspnea or fatigue. Deposition of amyloid fibrils in the atrium and the conduction system, including the atrioventricular (AV) node, may result in various rhythm abnormalities. These range from atrial tachycardias such as atrial fibrillation to AV conduction delays to heart blocks. In recent studies on the autopsied heart, amyloid deposition in the ventricles is found frequently; however, sustained ventricular tachycardias are not frequently reported. ^3^^–^^5^

Plasma cells produce monoclonal immunoglobulin light chains, which result in amyloid fibrils responsible for AL amyloidosis. Cardiac involvement in AL amyloidosis is attributed to two mechanisms. One is the extracellular deposition of amyloid fibril in the myocardium, and another involves direct cardiotoxicity from the fibril aggregates. ^3^ Although many organ systems are typically involved, cardiac involvement is the leading cause of mortality and morbidity in 50-70% of cases. ^6^ Diagnosis is achieved by detecting free light chains or monoclonal immunoglobulins in blood and urine. Treatment is through specifically directed chemotherapy. ^3^

Previously, ATTRv amyloidosis was considered to be of neurological importance and ATTRwt to be of systemic importance. This was because of the predominance of neurological symptoms, including neuropathies reported with ATTRv amyloidosis. ATTRwt amyloidosis was always known to be a culprit for cardiomyopathy. ^7^ However, with the recent recognition of the disease and the development of noninvasive diagnostic modalities, ATTRv amyloidosis has also been implicated in causing cardiomyopathy. ^7^ Despite these advances, there remains a vast number of undiagnosed cases of ATTR amyloidosis. Studies on valves replaced/removed by either transcutaneous or surgical approach have shown a 16-25% prevalence of ATTRwt deposits. ^8^ This has led to an increased focus on CA in pre-operative evaluation for transcatheter aortic valve replacement (TAVR). ^9^

In the absence of effective therapies for cardiac amyloidosis previously, diagnosis of AL and ATTR amyloidosis was not an area of focus. However, with the advent and availability of specific chemotherapy, early diagnosis and initiation of therapy are crucial. ^10^

## Diagnosis

Clinical clues are essential in leading clinicians toward thinking of amyloidosis. AL amyloidosis is associated with multisystem involvement. Thus, signs of nausea, vomiting, diarrhea, gastrointestinal bleeding for GI involvement, renal failure with albuminuria, and systemic symptoms of macroglossia are typical red flag symptoms. ATTRv amyloidosis may present with early neurological symptoms of neuropathies which may be diffuse or axonal. ^7^ ATTRwt amyloidosis may concern patients with bilateral carpal tunnel syndrome and lumbar stenosis, as such manifestations may predate cardiomyopathy by decades. ^9^

Electrocardiography is typically the first diagnostic study to be performed due to its easy availability. Low voltage QRS complexes have been known to be associated with cardiac amyloidosis, although recent data suggests it may be over-implicated. Almost 70% of patients with proven cardiac amyloidosis may not have low voltage and may have a standard or even high voltage meeting the criteria for left ventricular hypertrophy. ^11^ Atrial deposits can result in arrhythmias, and deposits in the conduction system resulting in heart block may represent other findings on the electrocardiogram. Pseuodoinfaction identified by pathological Q waves or QS waves on two consecutive leads is seen in patients with AL amyloidosis. If present, the study demonstrates worse outcomes of AL amyloidosis. ^12^

Echocardiography has been an essential tool for the evaluation of patients with symptoms of heart failure. Unexplained LV thickness >12 mm, along with grade 2 or worse diastolic dysfunction and reduced tissue doppler velocity, can meet echocardiographic criteria; however, these need to be coupled with biopsy or nuclear imaging. ^13^ LVEF has been previously used to diagnose and monitor patients with decline associated with disease progression. It is noted that LVEF declines later, and thus, it does not accurately predict the worsening of the disease. ^14^ Global longitudinal strain (GLS) has developed as an emerging tool to predict diagnosis and outcome accurately. Relative apical sparing of GLS resulting in a bull’s eye pattern of apical sparing is strongly suggestive of CA, differentiating from other causes of cardiomyopathy. ^14^ GLS has proved to be superior to LVEF in diagnosis, risk stratification, and decision-making amongst patients with AL amyloidosis. ^15^

Cardiac magnetic resonance (CMR) utilizes its intrinsic property to differentiate normal myocardium from pathologic through T1 and T2 signals. This, when coupled with late gadolinium enhancement (LGE) and extracellular volume (ECV) measurement, vastly improves the diagnostic ability for CA. ^16^ Recent studies challenge the typical LGE pattern in CA of global subendocardial enhancement. Progression of LGE pattern from subendocardial to transmural has been recently reported. ^16^ CMR was also studied for its ability to differentiate between AL and ATTR amyloidosis; however, it was not found to be sensitive or specific. ^10^

Nuclear imaging with bone scintigraphy and single photon emission computed tomography (SPECT) is widely available. 99m Technetium-pyrophosphate (PYP) is increasingly used to diagnose ATTR amyloidosis in the United States. It is also now recognized as confirmatory in the absence of cardiac biopsy. ^1^ 99mTc-hydroxydiphosphonate (HMDP) and 99mTc-DPD are other radiotracers used in Europe, depending on availability. Heart to contralateral lung ratio (H/CL) evaluated 1 hour after uptake quantifies cardiac uptake. Visual uptake is reported by the Perugini grading system, with grade 0 reported with no cardiac uptake, grade 1 reported with mild cardiac uptake compared to rib, grade 2 with equal uptake, and grade 3 with higher cardiac uptake compared to rib. ^16^ While studies have shown that grade 2 and 3 uptake and H/CL ratio >1.5 are sensitive for ATTR amyloidosis, mild uptakes are also noted in about 40% of cases with AL amyloidosis. ^17^ Recently, the H/CL ratio has been challenged. Mitral annular and aortic valve calcifications can cause increased uptake while myocardial scars can cause decreased uptake. ^18^ Blood-blood imaging (PYP in blood pool in RV and LV cavity) can cause false positives. Therefore SPECT evidence for PYP uptake is considered a diagnostic cornerstone. ^19^

**Figure 1.**
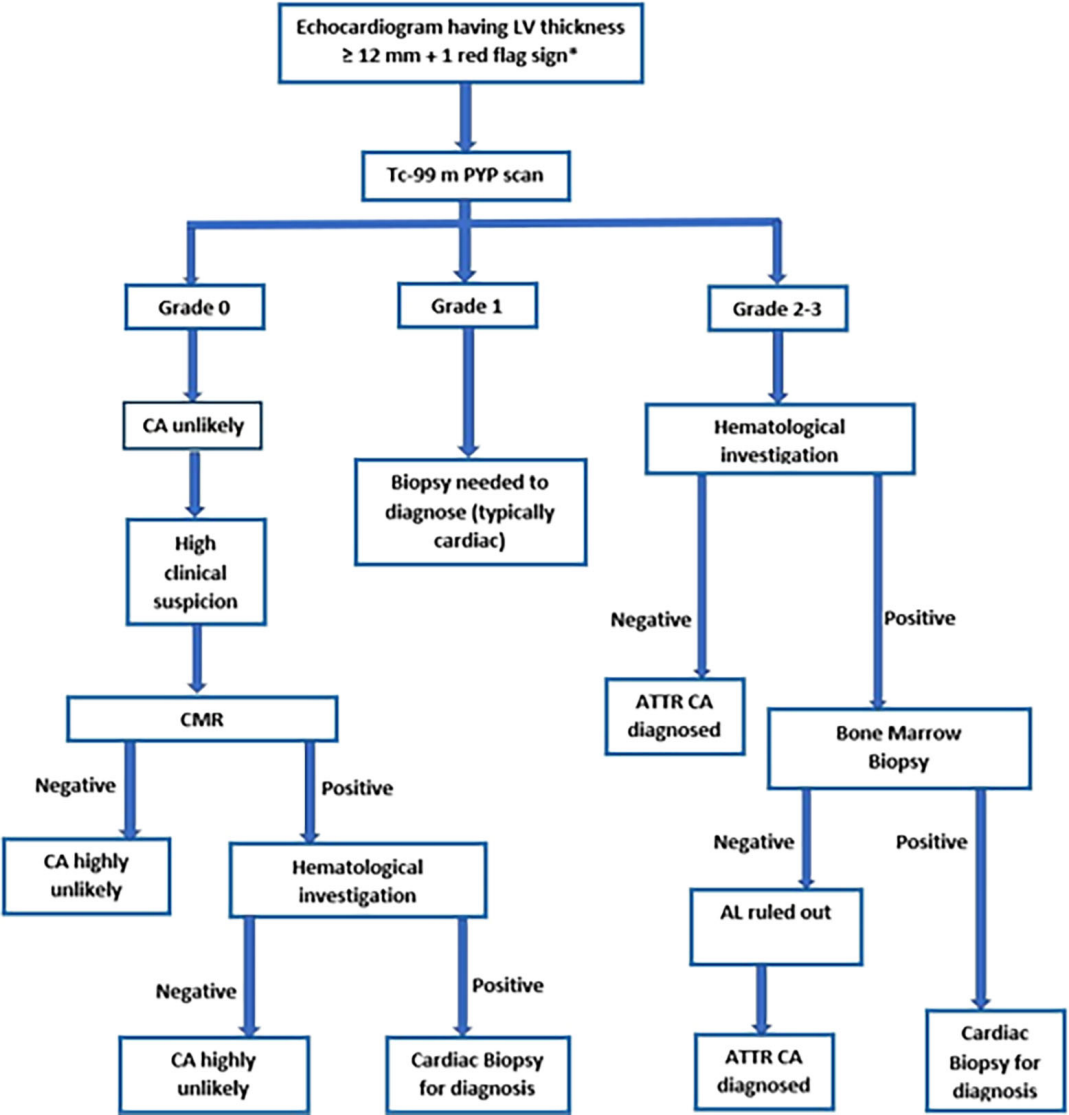
Patient selection and study design. LV-left ventricle, Tc-99m PYP-Technetium-99m pyrophosphate, CA-cardiac amyloidosis, CMR-cardiac magnetic resonance, ATTR- transthyretin amyloidosis, AL-amyloid with light chain *Red flags - heart failure ≥65 years, aortic stenosis ≥65 years, hypotension or normotensive if previously hypertensive, sensory involvement, autonomic dysfunction, peripheral polyneuropathy, proteinuria, skin bruising, bilateral carpal tunnel syndrome, ruptured biceps tendon, subendocardial/transmural late gadolinium enhancement or increased extracellular volume fraction, reduced longitudinal strain with apical sparing, decreased QRS voltage to mass ratio, pseudo Q waves on ECG, atrioventricular conduction disease, possible family history.

Given AL amyloidosis's different management and prognosis, it is important to rule it out with the help of urine and blood immunoglobulin light chains. Once AL amyloidosis is ruled out, grades 2 and 3 uptake become more than 90% sensitive and 98% specific for ATTR CA when coupled with positive SPECT images. ^20^ With the availability of such a test, obtaining a PYP scan in patients with LV thickness ≥12 mm on echocardiogram and any one red flag sign is recommended. ^13^ We utilize the algorithm as noted in Figure 1 for the diagnosis of CA.

Position emission tomography (PET) has been studied and suggested to have a role in diagnosis and therapeutic monitoring. ^21^ However, studies with a direct comparison between SPECT and PET have demonstrated the superiority of SPECT over PET in terms of sensitivity and specificity for the diagnosis of ATTR CA. ^22^

## Treatment of cardiac complications

Treatment of cardiac amyloidosis focuses on managing cardiac complications and preventing the worsening of underlying organ failure. The most common complication encountered clinically is heart failure. Typically, it presents as HFpEF. Management strategies with usual heart failure medications may prove harmful in CA. ACE inhibitors/ARBs may aggravate orthostatic hypotension secondary to activation of the renin-angiotensin-aldosterone system due to autonomic dysfunction. ^23^ Beta-blockers can also precipitate hypotension by decreasing heart rate and contractility; however, a recent study from Italy has demonstrated that beta-blockers may be tolerated for the management of co-morbidities even in the presence of CA. ^23^^,^^24^ Calcium channel blockers are implicated in binding to amyloid fibrils which may result in cytotoxicity. If treated with digoxin, CA is already prone to arrhythmias due to amyloid deposition in the atrium, which can increase fatal arrhythmias. ^23^ Treatment should be diuretics which help relieve congestion as needed. Loop diuretics have been used for an extended period in CA; however, recent data suggest vasopressin receptor antagonists may be more suitable for achieving balanced euvolemia and avoiding the risk of hypotension with loop diuretics. ^25^ In addition, recent emperor preserved and deliver trials have shown the benefit of sodium-glucose cotransporter 2 inhibitors in patients with HFpEF in decreasing hospitalizations and are now incorporated in HFpEF management with 2a evidence. Although specific studies on patients with CA are lacking, data from these trials can be extrapolated in this specific circumstance. ^26^^,^^27^

Atrial fibrillation is a known complication of CA; however, its management has been of interest recently. Rate control medications such as beta-blockers and calcium channel blockers are relatively contraindicated, as noted above. Rhythm control strategies may be appropriate, and amiodarone is the antiarrhythmic drug of choice. Electrical cardioversion is also attempted; however, it is essential to rule out LA thrombus, and the recurrence rate of atrial fibrillation is high at 51%, as noted in a recent study. ^28^ A similar value holds for catheter ablations, with a recent study demonstrating an 80% recurrence rate at two years. ^29^ Anticoagulation with NOACs is indicated irrespective of the CHA2DS2-VASc score because of the risk of thrombus formation in CA due to its inherent risk of thrombogenicity beyond known risk factors. ^30^

Coronary artery disease (CAD) in patients with CA presents various issues regarding management. It is possible that in a person with CAD, it may mask the symptoms of CA. Ischemic cardiomyopathy resulting in heart failure symptoms may prolong the diagnosis of cardiac amyloidosis. An issue arises when medical therapy with beta-blockers and ACEI/ARBs are indicated for ischemic cardiomyopathy but avoided in CA. Patients with triple vessel CAD with known CA need to be considered for percutaneous coronary intervention (PCI) versus coronary artery bypass graft (CABG). The benefits of CABG over PCI in triple vessel disease with diabetes are typically seen in the long run. ^31^ ATTR CA has a better prognosis than AL amyloidosis; however, that as well is about 3-5 years. ^3^ A decision that will need to be addressed in this scenario is whether the short-term risk of CABG outweighs its long-term benefits, given prognosis is not prolonged to reap the benefits of CABG. Heart block is a known complication of CABG, and the risk may be very high in patients with CA. Case reports have shown increased morbidity in patients with CA undergoing CABG. ^32^ Further studies looking specifically at the question of PCI versus CABG in patients with CA and triple vessel CAD are needed. ^33^

Aortic stenosis (AS) and its association with CA are well known now, with studies showing the incidence of CA in AS patients undergoing valve replacement as high as 29%. ^34^ It is still unclear precisely if CA contributes to AS or if they are independent and association is merely age-related. Deposition of amyloid fibrils on the valve resulting in AS argues for CA contributing to AS. Recent data suggest that combined AS-CA is associated with worse 1-year-mortality compared to lone AS. ^35^^,^^36^ Previously, ATTR amyloidosis therapies were not vastly available and approved. Now with TTR stabilizer and silencer therapy available, various new areas to explore are available. Two questions are critical to avoid such an increased risk of heart block; 1) Whether patients with severe AS undergoing TAVR should be routinely tested for CA? 2) If they are tested and found to have ATTR CA, should they receive a trial of TTR stabilizer/silencer medication prior to valve intervention? Data regarding this is lacking at this point.

Pacemaker implantation is seen frequently in patients with CA who develop conduction disorders. These are the patients who develop symptomatic bradycardia or high-degree atrioventricular block. The question that arises with this is, can pacemaker implantation be considered in high-risk individuals for primary prevention? There has been sparse data regarding the role of permanent pacemaker implantation (PPM) for primary prevention and the optimal time to pursue such a step. A recent study by Milner *et al.* on patients referred for liver transplants tried to answer this question. Data did not suggest improved mortality or morbidity in patients who received a PPM before liver transplantation for prophylaxis. ^37^ A recent study by Porcari *et al.* noted that 8.9% of patients with the diagnosis of CA needed PPM implantation within 3 years of diagnosis. ^38^ Thus, until further data is available, it is reasonable to reserve pacemaker placement for secondary prevention.

Cardiac resynchronization therapy (CRT) has been previously suggested to be used for patients in whom a high-paced burden is expected who are undergoing pacemaker placement. ^13^ Traditionally indicated for patients with LVEF <35% with left bundle branch block and QRS >150 ms; theoretically, it also plays a role in CA. Infiltrative cardiomyopathies in which RV pacing leads to LV systolic dysfunction, and when AV conduction block is associated with LV dysfunction, CRT may be indicated. Detailed data on CRT in patients with CA is lacking. A recent study on major cardiovascular events and outcomes after CRT implantation in CA patients has not shown any benefit and has suggested that it may be associated with worsened HF symptoms and hospitalization. Compared to dilated cardiomyopathy, CRT had much lower response rates in patients with CA. ^39^

An implantable cardiac defibrillator (ICD) has a role in the primary prevention of sudden cardiac death in patients with LVEF <35%. Electromechanical dissociation is likely the cause behind SCD in CA. ICD placement is recommended in CA for secondary prevention after the patient has developed sustained ventricular tachycardia. ^40^ However, its role in the primary prevention of SCD in patients with CA is not established. ^41^ Nonetheless, recent case reports have opened a new discussion area in this matter. Patients with cardiac amyloidosis who develop non-sustained ventricular tachycardia may not qualify for ICD placement just yet but do qualify for an electrophysiological study (EPS) to identify a potential area of the arrhythmogenic substrate for ventricular tachycardia. ^42^ Further studies are needed for risk stratification and establishing guidelines regarding primary ICD in such patients.

Cardiac transplantation has been performed for decades in patients with cardiac amyloidosis, particularly AL amyloidosis. However, outcomes were not favorable previously. This was secondary to extracardiac involvement and limitations of chemotherapy options. With the advent of advanced chemotherapy given even after cardiac transplantation and better diagnostic techniques allowing extracardiac detection, proper candidates are being chosen for cardiac transplantation associated with improved outcomes. ^43^

Mechanical circulatory support (MCS) has not been studied adequately in CA. Being restrictive cardiomyopathy, to implant assist devices in CA with small cardiac cavities is technically challenging. If selective left ventricular assist devices are placed, it increases the risk of right-sided heart failure. Also, considering patients with CA being on active immunosuppressive therapy, the risk of infection needs to be considered. Despite these factors, there has been recent data to suggest a role of MCS in patients with left ventricular end-diastolic diameter >46 mm. Even if performed as a bridge to transplantation, MCS is associated with increased survival. ^44^

## Disease-modifying treatment

AL amyloidosis is managed with various chemotherapy agents. Oral melphalan with steroids has been used for treatment for an extended period. Autologous stem cell transplant and melphalan became the mainstay of treatment in the 1990s and remained robust treatment options. However, the introduction of proteasome inhibitors revolutionized the treatment of AL amyloidosis with the enteral agent of bortezomib. ^45^ In a recent study, bortezomib is shown to have a significant hematological response and improved survival. ^46^ Various regimens have been studied, but no regimen is found to be superior to others. ^47^ Newer therapies with anti-CD38 human IgG monoclonal antibody agents daratumumab and isatuximab are currently under investigation, with preliminary data showing better short-term outcomes. ^48^

ATTR amyloidosis therapies are aimed at two mechanisms. One is to silence the TTR gene, and the other is to stabilize the TTR tetramers preventing them from forming monomers and thus constituting amyloid fibrils. TTR stabilizer includes Tafamidis and Diflunisal, showing promising data with substantially improved long-term outcomes in recent studies. ^49^ Patients with ATTR CA treated with Tafamidis have had reduced CV-related hospitalizations and length of stay in the hospital. ^50^ The most significant barrier to tafamidis being widely used is its cost-effectiveness. Even with insurance, it is estimated that the cost of production of tafamidis may need to be reduced by >90% for it to be cost-effective. ^51^^,^^52^

Gene silencer therapy with Patisiran has been studied in ATTRv and has shown significant improvement in neurological symptoms in patients with ATTRv amyloidosis after liver transplantation. ^53^ Another gene silencer medication Inotersen has shown similar improvement. ^54^ Many patients with ATTRv amyloidosis may have a mixed phenotype of cardiomyopathy and polyneuropathy. These patients can be considered for either TTR stabilizer or gene silencer therapies. Current practice and guidelines recommend selecting medication based on the predominance of cardiac or neurological phenotype. However, data to support this approach is not conclusive. Given their mechanism of action, gene silencer medications may be even more effective than TTR stabilizer medications in managing ATTR CA.

Most recently, targeted delivery of gene-editing therapy with the Cas9 endonuclease (CRISPR-Cas9) system to the hepatocytes is being studied. This is a single infusion and reduces the production of transthyretin by the hepatocytes. ^55^ Clinical trials are ongoing, focusing on new therapies such as vutrisiran, AG10, doxycycline, green tea, and monoclonal antibody NNC6019-0001 for their role in managing ATTR CA. ^56^

## Conclusion

Many recent advances have been made in diagnosing and managing cardiac complications of amyloidosis and disease-modifying treatment. Bone scintigraphy has played a pivotal role in early, non-invasive diagnosis coupled with disease-modifying therapy with medications such as Tafamidis and Bortezomib have changed the perception of CA. With better diagnostic capabilities, cardiac complications need to associate with CA and be managed based on specific guidelines obtained from data of CA patients. This opens new doors for research questions. Management of triple vessel CAD with co-existent CA with PCI versus CABG, routine PYP scan prior to TAVR, a trial of Tafamidis prior to valve intervention, ICD for primary prevention of SCD in CA after EP study, MCS in patients with adequately sized cardiac chambers are few of such questions which we have raised and hypothesized in this article.

## Author contributions

MR prepared the manuscript with the help of LS, AJ, SP, JT, KUC, DP, and RD. SS and KS are the senior authors who helped revise the manuscript and reviewed it for intellectual content.

## Data Availability

No data are associated with this article.
