# Supplementary material for: A review of recent advances in the diagnosis of cardiac amyloidosis, treatment of its cardiac complications, and disease-modifying therapies
Source: F1000Res. 2023 Feb 20;12:192. [Version 1] doi: 10.12688/f1000research.130285.1 (PMC9995736; doi:10.12688/f1000research.130285.1)
Supplement: Peer review history

# Peer review: Reviewer response for version 1

**DOI:** 10.5256/f1000research.143029.r164944

**Published:** 2023-03-08

**Authors:** Bhavi Trivedi

**Affiliations:** 1 Department of Internal Medicine, Texas Tech University Health Sciences Center El Paso, El Paso, TX, USA

This is a comprehensive review of cardiac amyloidosis. Figure 1 was a good overview. A good number of resources were cited. The authors provided a detailed oriented review of cardiac amyloidosis and advances in the field. The information provided is thorough and has efficiently summarized the management strategies and disease modifying therapies. I did not answer "no" or "partly" to any questions.

Is the review written in accessible language?

Yes

Are all factual statements correct and adequately supported by citations?

Yes

Are the conclusions drawn appropriate in the context of the current research literature?

Yes

Is the topic of the review discussed comprehensively in the context of the current literature?

Yes

Reviewer Expertise:

Internal Medicine

I confirm that I have read this submission and believe that I have an appropriate level of expertise to confirm that it is of an acceptable scientific standard.

# Peer review: Reviewer response for version 1

**DOI:** 10.5256/f1000research.143029.r164947

**Published:** 2023-03-06

**Authors:** Apoorva Kondapally

**Affiliations:** 1 Willis-Knighton Medical Center, Shreveport, LA, USA

An excellent report in reviewing the key aspects of cardiac amyloidosis focusing on recent advances and current literature. All statements appear to be factual, and the language is appropriate.

 It has detailed recommendations of recent advances in management with adequate and appropriate citations and the conclusions are made in accordance with the current literature.

Is the review written in accessible language?

Yes

Are all factual statements correct and adequately supported by citations?

Yes

Are the conclusions drawn appropriate in the context of the current research literature?

Yes

Is the topic of the review discussed comprehensively in the context of the current literature?

Yes

Reviewer Expertise:

Areas of interest and research are endocrinology, immunology, neurology.

I confirm that I have read this submission and believe that I have an appropriate level of expertise to confirm that it is of an acceptable scientific standard.
